# Combination of Alcohol and EVOH as a New Embolic Agent: Midterm Tissue and Inflammatory Effects in a Swine Model

**DOI:** 10.1155/2020/8831060

**Published:** 2020-10-23

**Authors:** Jean-François Hak, Farouk Tradi, Mickael Bobot, Pauline Brige, Paul Habert, Sophie Chopinet, Aurélie Haffner, Gilles Soulez, Benjamin Guillet, Vincent Vidal

**Affiliations:** ^1^Diagnostic and Interventional Radiology Section, Department of Medical Imaging, University Hospital Timone APHM, 278 Rue Saint-Pierre, Marseille 13005, France; ^2^Aix-Marseille University, LIIE, Marseille, France; ^3^Aix-Marseille University, CERIMED, Marseille, France; ^4^Department of Nephrology, University Hospital Conception APHM, Marseille 13005, France; ^5^INSERM 1263, INRA 1260, C2VN, Aix Marseille University, Marseille 13005, Timone APHM, France; ^6^Department of Digestive Surgery, University Hospital Timone APHM, Marseille 13005, France; ^7^Department of Pathological Anatomy, University Hospital Timone APHM, Marseille 13005, France; ^8^Department of Radiology, Centre Hospitalier de L'Université de Montréal, Sherbrooke East, Montreal H2L 4M1, Montreal 1560, Canada; ^9^Department of Radiopharmacy, University Hospital Conception APHM, Marseille 13005, France

## Abstract

**Objective:**

To evaluate the vascular occlusion and midterm tissue toxicity properties of a combination of ethylene-vinyl alcohol (EVOH) (Squid 18®) (75%) and alcohol (25%)—Alco-Squid 18—in a swine model.

**Materials and Methods:**

Alco-Squid 18 (75% Squid 18® mixed with 25% alcohol) (AS18) was compared to embolization with 96% alcohol alone and to embolization with Squid 18® (S18®) alone. An arteriovenous malformation (AVM) model was created in group 1 (*n* = 2). Each AVM model was then embolized with AS18 or S18® alone with evaluation of a ratio between the volume of embolic agent divided by the volume of the AVM (evaluated by CT). For group 2 (*n* = 5), each agent was tested on three different kidneys (upper pole kidney artery). Pre- and postinterventional CTs, angiographies, blood alcohol content dosages, and histological studies (3 months postintervention) were performed.

**Results:**

AS18 has better distal distribution than S18® alone, both in the kidneys (mean capsule-S18® distance: 3.9 mm (±0.23) and mean capsule-AS18 distance: 2.3 mm (±0.11) (*p*=0.029) and in the AVM model. Histological exploration found a higher rate of tubular necrosis with AS18 compared with S18® alone and alcohol alone (3.78 ± 0.44 compared to 2.33 ± 1.22 (*p* = 0.012) and 1.22 ± 0.67 (*p*  < 0 .0001)). The blood alcohol content was negligible in all cases.

**Conclusion:**

AS18 can suggest a better distal sclerotic and embolic character as compared with S18® alone without systemic toxicity.

## 1. Introduction

Squid® and Onyx® are liquid embolic agents (LEAs), respectively, manufactured by Balt (Balt, Gland, Switzerland) and Medtronic (Medtronic, Irvine, California, USA) which are effective in the treatment of cerebrovascular lesions, chronic aortic endoleaks, other organ lesions, and peripheral nonneurologic bleeding. These are a mixture of ethylene-vinyl alcohol copolymer (EVOH), dissolved in dimethyl sulfoxide (DMSO), with added micronized tantalum powder, making the mixture visible under fluoroscopy. When these LEAs are in contact with blood or any aqueous solution, EVOH copolymer precipitates and solidifies itself into a spongy cast from the outside in, as a result of DMSO solvent diffusion. Even if these LEAs allow vascular occlusion, they are unable to induce an effective sclerotic effect on the target vessels, possibly resulting in recanalization and regrowth [1] Thus, clinicoradiological recurrence rates of arteriovenous malformations (AVMs) were observed after arterial embolization with EVOH alone in 6.9% and up to 18.2% of cases in histological analysis.

Alcohol (96% ethanol) is one of the most effective sclerosing and deeply penetrating LEAs. It acts by inducing cytotoxic damage and by thrombosis [2–4], resulting in fibrinoid necrosis [5].

Due to its low viscosity and poor visibility, the systemic migration of alcohol induces a considerable risk of nontarget embolization and potentially serious side effects (e.g., vasospasm, nerve damage [6], acute pulmonary hypertension [7], cardiac collapse [8], and skin necrosis [9]), with a mortality rate reported of 0.6%).

An ideal embolic liquid agent should possess both sclerotic and embolic characteristics without systemic toxicity [10].

Hamada et al. reported different studies about a mixture of EVOH-alcohol composed of 4 g EVOH, 60 g iopamidol with an iodine concentration of 300 mg/ml (Iopamiron; Nihon Schering, Osaka, Japan), and 36 g of 96% alcohol as a solvent. But this combination aimed to replace DMSO with alcohol and required multiple time-consuming stages of development [11–13].

A preliminary feasibility study evaluated the best concentration combination of an already known and widely used LEA (Onyx 18®) with a sclerotic LEA (96% alcohol) and reported that a concentration of 75% and 25%, respectively, was the best combination. This combination without multiple time-consuming stages of development could be a new effective scleroembolic LEA. But this first feasibility study did not evaluate midterm efficacy [14].

The aim of this work is to evaluate vascular occlusion and midterm tissue toxicity properties of the combination of 75% of EVOH (Squid 18®) and 96% alcohol (25%)—AS18—reported by Saeed Kilani et al. [14] in a swine model.

## 2. Materials and Methods

This work was conducted on a swine model. The animals were used in accordance with institutional and national guidelines for the care and use of animals. Seven Pietrain pigs with a weight of 40 ± 5 kg were used. This study received approval from the Animal Care and Ethics Committee (APAFIS#14851-2018042610172296).

All experimental evaluations were performed under general anesthesia. Animal preparation, anesthesia, and euthanasia were performed as described in the previous study [15].

### 2.1. Preembolization Preparation and Liquid Embolic Agents (LEAs)

#### 2.1.1. Endovascular AVM Model (Group 1)

An AVM model was created using rete mirabile (RM) as previously described [16]. RM are even and symmetrical organs composed of vascular follicles and thin capillaries. The animals were prepared 3 months before the experimental procedures in order to allow the AVM nidus (corresponding to both RM) to grow and to increase vascular endothelial growth factor (VEGF) concentration [16]. The proximal portion of the ascending pharyngeal artery (2 cm) on one side and the carotid artery on the ipsilateral side were embolized with coils to get this endovascular AVM model.

This model has true AVM angioarchitecture with an arterial compartment (only one ascending pharyngeal artery), a nidal compartment (complex of two RM), and a venous compartment (internal jugular veins).

#### 2.1.2. Liquid Embolic Agents (LEAs)

The combination of 75% of Squid 18® (EVOH) and 25% of 96% alcohol (Assistance Publique Hôpitaux de Paris APHP, Paris, France), or Alco-Squid 18 (AS18), was compared to embolization with 96% alcohol alone and to embolization with Squid 18® (S18) alone.

Prior to this study, injectability, precipitation, and radiopacity tests on a combination of EVOH and alcohol in the same proportions had already been performed in vitro and in vivo in a preliminary feasibility study [14].

S18® was shaken for 20 min using an agitator (Vortex-Genie, Scientific Industries, Bohemia, NY) to homogenize the tantalum powder in the suspension for all the procedures.

To obtain the AS18 combination, alcohol 96% (25%; 0.5 mL) was injected directly into the vial of Squid® (75%; 1.5 mL). Then, the vial was shaken for 20 min just before the embolization procedure.

### 2.2. In Vivo Embolization

#### 2.2.1. Embolization Procedure

A digital subtraction angiography (DSA) system (Fluorostar, General Electric Medical System, Minneapolis, USA) was used for radiological procedures. Percutaneous access was aseptically performed by femoral arterial ultrasound-guided puncture with the Seldinger method using a 6-French vascular introducer.

Catheterization of vascular targets was performed using an Envoy® 5F catheter (DePuy Synthes, Raynham, MA, USA) allowing access to the ascending pharyngeal arteries and kidney arteries.

DSAs were performed first (Visipaque 320 mg I/ml, GE), and then embolization was performed through a DMSO-compatible microcatheter (Marathon; Covidien/ev3 Neurovascular, Irvine, CA) after selective microcatheterization.

Before embolization with S18® or AS18, the microcatheter dead space was flushed with saline serum before being filled with an adequate volume of DMSO.

All procedures were performed by the same experienced interventional radiologist and in the same fashion. The embolization endpoint occurred when a complete occlusion of the artery was achieved on final DSA images.

#### 2.2.2. Animal Settings

  Group 1 (*n* = 2): to study the physicochemical behaviour of the combination into a vascular disease animal model, an AVM model was performed by embolization [16].
  Group 2 (*n* = 5): to study midterm tissue effects, upper thirds of kidneys were embolized with a superior segmental artery as the sole target artery to keep the animals alive and to allow to evaluate midterm effects. The duration of follow-up was 3 months to study the midterm effects of implanted products (International Organization for Standardization; ISO10993-6(18)).

The animals from group 1 were euthanized immediately after the embolic procedure.

The animals from group 2 were euthanized 3 months (M3) after the intervention.

In group 1 (*n* = 2), three months after the AVM model preparation, one animal was embolized with S18® (*n* = 1) and the other with AS18 (*n* = 1) through the ascending pharyngeal feeder artery of the AVM model. When reflux occurred at the tip of the microcatheter, the injection was paused for 30 seconds–2 minutes. Usually, reflux may occur several times prior to S18® advancing into an AVM. Once S18® or AS18 advanced into the AVM model, it was slowly and continually injected. When the S18® or AS18 cast stopped advancement, we used small and short pulses of injection until the angiographic endpoint occurred. The endpoint of the procedure was complete AVM model occlusion or S18® or AS18 reflux longer than 3-4 cm to the microcatheter without evidence of further advancement of LEA into the AVM model. During the LEA injection, DSA was performed to check the AVM flow.

In group 2 (*n* = 5), after each embolization procedure, verification of the absence of complication of two-thirds of the nonembolized kidneys was assessed with DSA and computed tomography (CT) scan with intravenous iodinated contrast injection (tissue enhancement, no off-target embolization). Each LEA (S18® and AS18) was tested three times in the five pigs (10 targeted kidneys) corresponding to three targeted kidney arteries per LEA. Among these 10 kidneys, one was not embolized and kept as a histological reference at M3. The microcatheter tip was immediately positioned at the first bifurcation of each target artery. DSA endpoint was defined by visualizing reflux at the proximal bifurcation (except for alcohol embolization; not radiopaque). For 96% alcohol embolization, 1 mL of LEA was used to occlude each target artery. If the DSA endpoint was not obtained with 1 mL of alcohol, a short pulse of 0.1 mL of alcohol was repeated with repeated DSA control between each pulse. A final DSA was performed through the catheter to confirm the complete occlusion with all the LEAs tested [17].

### 2.3. Study Goals

#### 2.3.1. Imaging Evaluations

For group 1, DSAs and contrast-enhanced CTs (Visipaque 320 mg I/ml, GE) (Discovery CT 750; GE Healthcare, Chicago, Illinois, US) were obtained for pigs immediately after the embolization procedure. In order to overcome the variability in size and morphology of RM between pigs, a ratio was calculated by dividing the volume of embolic product injected into the AVM model (data collected during the procedure) and the external volume of RM (measured with CT) [18].

For group 2, DSAs and contrast-enhanced CTs were obtained for all the pigs immediately after kidney embolization with S18®, AS18, and alcohol to evaluate embolization effectiveness (day 0; D0). DSAs and CTs were performed at 3 months (M3) to evaluate distal distribution, arterial recanalization, and kidney parenchyma.

Distal distribution of LEAs was evaluated in the coronal plane and transverse images of the embolized upper pole of the kidneys to determine the shortest distance between the visible embolic agent and the kidney capsule (except for alcohol; not radiopaque) [18].

All CT evaluations were performed by a radiologist blinded to the study arm.

#### 2.3.2. Blood Analysis

The systemic blood alcohol level was measured immediately after each complete embolization (confirmed with the last angiogram) and at the end of the embolization procedure (corresponding to the removal of the vascular introducer and the end of the anesthesia) using a venous catheter.

#### 2.3.3. Kidney Study

Animals from group 2 were sacrificed and surgically dissected 3 months after embolization [18]. Embolized organs were explanted for the histological study to evaluate distal distribution and sclerosing effect of the liquid agents on the endothelium. Postembolization tissue recovery was evaluated by kidney histology. The kidneys were then dissected into both the medullary and cortical parts, transferred into 4% buffered paraformaldehyde, and embedded in paraffin. Slides for each part (4 *μ*m thick) were prepared using a microtome (Microm, France) and stained with haematoxylin-eosin (HE) (AutoStainer, DRS 2000 Sakura). A qualitative and semiquantitative evaluation was carried out by an experienced anatomical pathologist blinded to the study arm as follows: (1) inflammatory parameters were evaluated using a semiquantitative scoring system (0 to 4), as previously described [19]: fibrin, arterial recanalization, haemorrhage, and cellular inflammatory parameters (fibroblasts, polynuclear, lymphocytes, macrophages, and giant cells); (2) tubular necrosis was assessed using a scale (from 0 to 4) designed to evaluate the degree of tubular necrosis and was defined as tubular dilatation and/or atrophy, inflammatory cell infiltrate, or cellular oedema, as previously described [20,21]. The scoring was performed by two independent trained pathologists blinded from the intervention.

### 2.4. Statistical Analyses

All statistical analyses were performed using SPSS 20.0 software (SPSS Inc., Chicago, IL). Quantitative data are expressed as the means ± SD and were compared using two-tailed nonparametric Mann–Whitney tests as data followed a nonnormal distribution. A *p* value of 0.05 was considered significant.

## 3. Results

### 3.1. General Observations

All embolization procedures were performed as planned. No technical failures or complications such as reflux, off-target embolization, or catheter occlusion were observed. The embolization endpoint was achieved in all kidneys. As demonstrated in DSA, the embolization endpoint was effective for occluding the vascular network in the upper pole in all study groups. The animals from group 2 did not show any clinical or behavioural deterioration during the 3-month follow-up.

### 3.2. Immediate Postembolization Results

#### 3.2.1. Depth Penetration in the AVM Model (Group 1)

**Figure 1:**
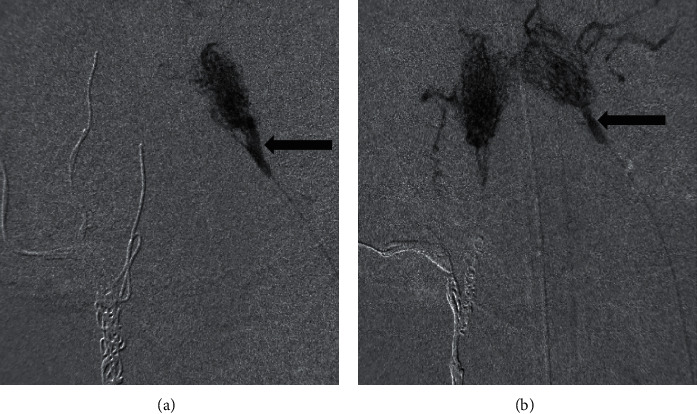
Embolization of the AVM model (group 1; *n* = 2). Black arrows corresponding to the tip of the microcatheter: (a) embolization with S18® alone (*n* = 1) (embolization limited to only one rete mirabile corresponding to half of the nidus AVM model); (b) embolization with AS18 (*n* = 1) (embolization of both rete mirabile corresponding to the whole nidus AVM model).

Each RM volume (*n* = 2) was evaluated with CT before embolization (2.33 and 1.75 mL). AS18 (*n* = 1) had a better depth penetration than S18® (*n* = 1) alone in the AVM models (*n* = 2). More AS18 (1.8 mL; ratio = 0.77) was injected than S18® (1 mL; ratio = 0.57) before objectifying angiographic endpoint. Embolization with S18® alone allowed to embolize one RM corresponding to the half of the AVM nidus model (Figure 1(a)) without reflux upstream of the distal portion of the microcatheter, whereas AS18 allowed the embolization of both RM, corresponding to the entire AVM nidus model (Figure 1(b)).

#### 3.2.2. Immediate Effectiveness and Depth Penetration of Embolization (Group 2)

**Figure 2:**
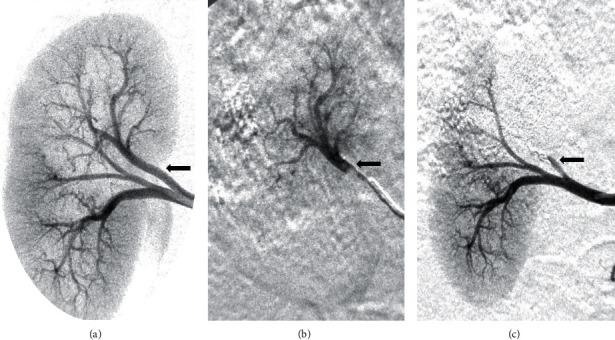
Example of right kidney DSA during embolization procedure: before (a), during (b), and after (c) embolization with AS18. Black arrows correspond to the target artery.

All kidney artery embolization procedures were considered successful when embolization endpoint occurred corresponding to a complete occlusion (Figure 2). AS18 had better distal distribution in the kidney as compared to S18® alone (average distance capsule-S18®: 3.9 mm (±0.23) and average capsule-AS18 distance: 2.3 mm (±0.11) (*p*=0.029). As a nonradiopaque LEA, embolization procedures using alcohol alone (96%), this CT measurement method using CT could not be performed.

#### 3.2.3. Alcohol Blood Level and Radiopacity

The blood alcohol level was negligible during all embolization procedures. Radiopacity from S18® was preserved when using AS18.

### 3.3. Midterm Postembolization Results

#### 3.3.1. Midterm Effectiveness of Embolization

The 3-month CT after the embolization procedure showed no complications, such as abscess, urinoma, or perirenal infiltration. Kidney hypotrophy was observed, corresponding to the embolized kidney area with an embolic agent located immediately below the kidney capsule. The CT evaluation at 3 months of the embolized kidneys from the different pigs did not show any recanalization for the different LEAs.

#### 3.3.2. Histological Study

The tubular damage was more important with AS18 (tubular necrosis score: 3.78 ± 0.44) as compared to S18® alone (2.33 ± 1.22, *p* = 0.012) or alcohol alone (1.22 ± 0.67, *p* < 0.0001). Kidney inflammation was not significantly higher with AS18 compared with S18® alone (fibroplasia: *p* = 0.726; polynuclear: *p* = 0.326; lymphocytes: *p* = 0.685; giant cells: *p* = 0.744; granulomatous infiltration: *p* = 0.533; extravasation: *p* = 0.999). The study of kidney parts embolized with alcohol found no inflammation and only slight tubular necrosis.

**Figure 3:**
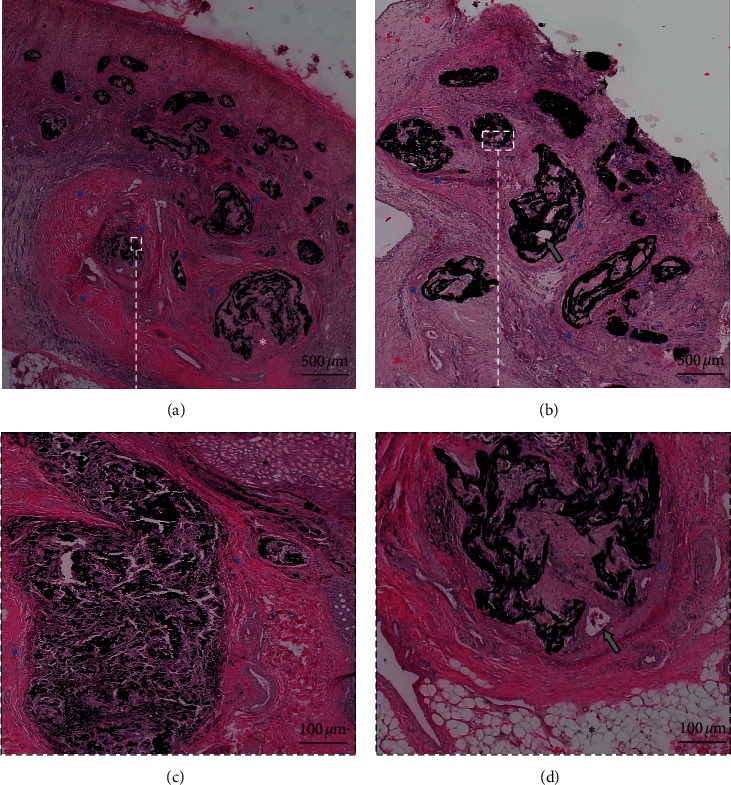
Corticomedullary kidney histological analysis with light microscopy: (a, c) after embolization with AS18, infiltration of arterial lumens by granulomas (giant cells, fibroblasts, polynuclear cells, and lymphocytes) (blue asterisks) without any recanalization (white asterisk); (b, d) after embolization with S18®, infiltration of arterial lumens by granulomas (blue asterisks) and recanalization of embolized arteries (grey arrows). (a, b): ×20 magnification (c, d): ×100 magnification.

Histological arterial recanalization after embolization with S18® alone was found in 25.5% of cases as compared to 11.1% with AS18 (*p* = 0.049) and 0% with alcohol alone (*p* > 0.99) (Figure 3).

## 4. Discussion

This experimental study compared embolization with AS18, S18®alone, and 96% alcohol alone. According to the histopathological analysis, the midterm tubular damage was more important with AS18 (tubular necrosis score: 3.78 ± 0.44) as compared to S18® alone (2.33 ± 1.22, *p* = 0.012) or alcohol alone (1.22 ± 0.67, *p*  < 0 .0001). Besides, the results highlight a better distal distribution with the AS18 combination as compared to S18® alone. Thus, AS18 alone had a better depth penetration than S18® alone in the AVM models (group 1) and in kidney arteries (group 2) before objectifying angiographic endpoint. These results confirm the previous preliminary feasibility study about distal LEA distribution [14]. However, no difference was found on kidney inflammation, but this may be due to our limited sample size.

In our study, histological arterial recanalization after embolization with AS18 was found in only 11.1% of cases as compared to 25.55% with S18® (*p*=0.049). Histological recanalization after embolization has been described in 18.2% of embolization with EVOH alone [20].

For kidneys' embolization using 96% alcohol, only a few amounts (1.4 ± 0.3 mL) were needed to get a total occlusion of the target artery. In our study, after embolization with 96% alcohol, there was no inflammation but only slight tubular necrosis. Yet, even with a low dose of alcohol, it is known that alcohol alone is one of the most effective sclerosing and deeply penetrating agents [2–4]. Our results are probably explained by the better viscosity of AS18 compared to 96% alcohol. Indeed, studies have shown that embolization with a more viscous mixture of alcohol and iodine is more sclerosing than embolization with alcohol alone [22]. The mixture of alcohol and iodine would also allow better retention of the mixture within the target arteries [23].

The tubular damage rate after embolization with AS18 was significantly higher as compared with S18® alone and alcohol alone. In the initial phase of a kidney ischemia-reperfusion process, the lesions involve a sterile inflammatory response that contributes to tubular cell damage. Subsequently, damaged necrotic tubular cells act as enhancers of the inflammatory response by damage-associated molecular pattern not related motifs, which trigger an influx of various inflammatory cells into the kidney [24,25].

Histological analysis was not performed for animals from group 1 because inflammatory parameters could not be observed immediately after the embolization [13].

Hamada et al. [11–13] evaluated a mixture of EVOH, iodine, and alcohol as a new LEA for AVM embolization, but our study focused on another combination, Squid 18® (5.3% EVOH, DMSO, and 30% of tantalum powder) associated with 96% alcohol. The combination from Hamada et al. aimed to replace DMSO with alcohol and required multiple time-consuming stages of development: (1) it needed to be dissolved by heating the components to 80°C for 30 minutes because at room temperature EVOH precipitates in the iopamidol/alcohol mixture; (2) it needed to be sterilized for 20 minutes in a steam autoclave and then to be rewarmed to approximately 80°C for at least 5 minutes before use. Our combination only needs 96% alcohol to be injected directly into the vial of Squid® and then to be shaken just before the embolization procedure to homogenize the tantalum powder.

Our findings are of interest as they suggest that our Alco-Squid has many advantages: it appears to be a safe, radiopaque, and easy to use LEA without target artery recanalization.

Alcohol blood levels were negligible for all of our interventional procedures, even in procedures using alcohol alone. This phenomenon could be explained by the small amount of alcohol used for all the embolization procedures.

There were some limitations in our study. First, the limited number of animals included remains low, with 1.5 pigs per embolic agent. This work was conducted on swine models because of its anatomical similarities with humans [26]. This model allowed to use the interventional radiology equipment used in humans and to get enough kidney tissue for histological analysis.

## 5. Conclusion

AS18 can suggest a better distal sclerotic and embolic character as compared to S18® alone without systemic toxicity in a swine model.

## Data Availability

The data used to support the findings of this study are included in the article.

## Abbreviation

AVM: Arteriovenous malformation
LEA: Liquid embolic agent
CT: Computerized tomography
DSA: Digital subtraction angiography
EVOH polymer: Ethylene-vinyl alcohol
DMSO: Dimethyl sulfoxide
ISO: International Organization for Standardization
RM: Rete mirabile
VEGF: Vascular endothelial growth factor.
